# Correlation analysis between positivity rate of immunoglobulin G antibodies against pertussis toxin among community-based populations and reported pertussis incidence in Shandong, China: a seven-year seroepidemiological study

**DOI:** 10.1186/s12879-025-11802-9

**Published:** 2025-10-24

**Authors:** Yan Zhang, Manshi Li, Lei Feng, Zexin Tao, Xinyu Yuan, Guifang Liu, Aiqiang Xu, Li Zhang

**Affiliations:** Division of Expanded Immunization Program, Shandong Provincial Center for Disease Control and Prevention, Shandong Academy of Preventive Medicine, Shandong Provincial Key Laboratory of Infectious Disease Control and Prevention, No. 16992 Jingshi Road, Jinan, 250014 P. R. China

**Keywords:** Pertussis, Seroepidemiology, Antibodies against pertussis toxin, Estimated infection rate

## Abstract

**Background:**

The reported annual incidence of pertussis in China has shown a marked increase over the past decade, but it may be still underestimated. The purpose of this study was to understand the seroepidemiologic characteristics of pertussis in community-based populations and to assess the level of pertussis infection in the population.

**Methods:**

Between 2017 and 2023, one or two cities in each of the eastern, central and western regions of Shandong Province were selected as survey sites, and a total of six age groups of healthy individuals were enrolled by multistage stratified random sampling to carry out the survey. The serum level of pertussis toxin (PT) IgG antibody was quantified by indirect enzyme-linked immunosorbent assay.

**Results:**

A total of 9,238 subjects were enrolled, and the mean positive rate of PT-IgG antibody was 8.05% (95%CI: 7.50%~8.60%), with the highest in 2019 (10.70%, 95% CI: 9.19%~12.21%) and the lowest in 2020 (6.32%, 95% CI: 4.98%~7.66%). The highest positive rate was found in the age group of less than 3 years old (11.46%, 95%CI: 9.87%~13.05%), and the lowest rate was found in the 3 ~ 5 years group (5.40%, 95%CI: 4.28%~6.52%). There was a significant difference in the positive rate between age groups (χ^2^ = 43.098, *p* < 0.001). Comparison of trends in recent infection rates and reported incidence rates in the population, that showed a very strong linear correlation in the annual distribution (*r* = 0.821, *p* = 0.023), and an extremely weak linear correlation in age distribution (*r* = 0.086, *p* = 0.872). Estimated infection rates (/100,000) among people aged ≥ 3 years ranged from 5,257 (95%CI: 3,918 ~ 6,596) to 24,449 (95%CI: 22,157 ~ 26,740) by years, with estimated infection rates in eastern region (31,544.44-fold) and in older age group (292,340.00-fold for ≥ 20 years old group, 216,388.89-fold for 17 ~ 19 years old group) differed significantly from the reported incidence rate.

**Conclusions:**

The annual distribution trend of reported pertussis incidence rate aligns with the infection rate observed among community populations, the actual level of infection is likely to be seriously underestimated. Therefore there is a need to emphasize surveillance and consider additional booster immunizations for adolescents and adults.

**Supplementary Information:**

The online version contains supplementary material available at 10.1186/s12879-025-11802-9.

## Background

 Pertussis, a respiratory infectious diseases caused by *Bordetella pertussis*, continues to be a significant contributor of morbidity and mortality among infants worldwide, despite the existence of vaccines for more than eight decades. The introduction of whole-cell and acellular pertussis vaccines as part of childhood immunization strategies has undoubtedly resulted in a significant decrease in reported cases of pertussis and related fatalities among children. Nevertheless, since the 1980 s, pertussis has reemerged in numerous countries with established, extensive vaccination programs [1]. The World Health Organization (WHO) estimates a global incidence of approximately 24.1 million pertussis cases, with 90% occurring in developing countries [2].

In China, before the introduction of the pertussis vaccine, the annual incidence of pertussis was reported to be between 100 and 200 cases per 100,000 individuals [3]. The inclusion of combined diphtheria, tetanus, whole-cell pertussis vaccines (DTwP) into the Expanded Programme on Immunization (EPI) since 1978 has led to a significant reduction in the incidence of pertussis. However, because of the frequent adverse reactions linked to the DTwP vaccine, it was replaced by combined diphtheria, tetanus, acellular pertussis vaccines (DTaP) in 2007. Even with a DTaP vaccination coverage rate surpassing 99%, surveillance data has indicated a 25-fold increase in reported pertussis cases in 2023 as compared to 2009 [4].

Although there has been a marked increase in the annual incidence of pertussis over the past decade in China, it is widely believed that the reported incidence still underestimates the true scale of the disease. The current national diagnostic criteria for laboratory-confirmed pertussis cases in China require either a positive culture or a four-fold increase in the concentration of pertussis toxin (PT) IgG (PT-IgG) from the acute to the convalescent phase, and PCR technology was not included. Consequently, the majority of reported cases have predominantly relied on clinical symptoms for diagnosis. This approach may not accurately reflect the true epidemiological burden of pertussis, since milder clinical presentations of the infection were often not recognized or diagnosed. This may be particularly relevant for pertussis infections in teenagers who have been vaccinated and adults with well-established immune system, as they may act as important reservoirs for transmission to unvaccinated or partially protected infants and young children [5, 6].

Shandong Province, with a population over 100 million, initiated an enhanced pertussis surveillance program since 2016, proactively adopting advanced surveillance measures, including laboratory confirmation through the detection of PT-IgG and the utilization of PCR for ascertainment of infectious cases, ahead of other regions nationwide. This enhanced surveillance mechanism has also partly contributed to a heightened diagnostic awareness among physicians for pertussis. Since then, the reported incidence of pertussis in Shandong Province has seen a 4-fold increase from 2016 to 2018. Despite the notable increase in reported cases, the incidence among individuals aged 20 and above maintained a relatively low level, ranging from 0.02 to 0.14 per 100,000 individuals per year [7]. Although the current disease surveillance system in Shandong Province has shown improvements, it still faces challenges regarding its activity and sensitivity. Therefore, further research is essential to accurately understand the true epidemiological landscape of pertussis in the province.

A seroepidemiological survey could be an enhanced surveillance activity employed in pertussis epidemiology and outbreak assessment, which has been adopted and reviewed in various international contexts [8, 9]. In this study, we conducted a seven-year continuous seroepidemiological survey among randomly selected populations from 2017 to 2023, aiming to ascertain the epidemiological characteristics and infection status of pertussis in China and providing evidence that can inform the refinement of current immunization strategies and preventive interventions.

## Methods

### Study design and sample collection

Based on geographical distribution, Shandong Province was divided into three distinct regions, eastern (Qingdao, Yantai, Weifang, Weihai, and Rizhao), central (Jinan, Zibo, Dongying, Tai’an, and Linyi City) and western (Zaozhuang, Jining, Dezhou, Liaocheng, Binzhou, and Heze City). Annually, one or two cities from each region were randomly selected using the method of computer-generated random numbers, and within these cities, the same random selection process was applied to identify one county (district) as the survey sites. The determination of the required sample size for this study was based on the formula *n* = Z^2^P(1-P)/ε^2^. P represented 50% of pertussis seropositivity with a relative permissible error (ε) of 3% and a confidence interval of 95%. When the ratio of lost follow-up of 10% was taken into account, a total of 1174 participants were needed to be enrolled per year. Participants enrolled in the study were healthy individuals who didn’t exhibit symptoms of respiratory infection, had not used antibiotics in the three months preceding the survey, and had been residing at the survey site for at least 12 months. Participants with a primary or secondary immunodeficiency disease and those who had received blood, blood components, or immunosuppressive agents within the 6 months prior to enrollment were excluded. The participants were stratified into six age-based subgroups (< 3, 3 ~ 5, 6 ~ 12, 13 ~ 16, 17 ~ 19, and ≥ 20 years). Within each age group in one site, 30 to 50 individuals were random selected, ensuring a balanced gender distribution in a ratio of 0.9 ~ 1.1. Serum specimens were collected from each participant, and demography and epidemiology information, including name, gender, age, residential area, medical history, and vaccination status of pertussis, were meticulously recorded in the questionnaire (Zhang Y: Questionnaire of seroepidemiological survey, unpublished) specifically designed for this study. To guarantee the precision and uniformity of data collection, the questionnaire was crafted through relevant literature reviews, interviews among study population, and multiple rounds of content validity evaluations by experts. Prior to conducting the survey, interviewers were properly selected, briefed, and trained, and a small sample of our target population was selected to pilot and evaluate. A total of 9,238 datasets were collected from 2017 to 2023.

### Laboratory testing

Specimens were assayed for PT-IgG utilizing the enzyme-linked immunosorbent assay (ELISA) method at the laboratory of Shandong Provincial Center for Disease Control and Prevention. A commercial kit (EUROIMMUM Medizinische Labordiagnostika AG, Germany) was used, with strict adherence to the manufacturer’s protocols. Antibody activities were quantified in International Units/mL (IU/mL) and referred to the WHO international standard for human pertussis antiserum (NIBSC 06/140). Currently, there are no established cut-off values for PT-IgG that serve as a definitive indication of *Bordetella pertussis* infection in China. For this study, through integrating the guidelines provided by the ELISA kit manufacturer for interpreting results, along with findings from previous research [10, 11], we defined PT-IgG concentration of ≥ 40 IU/mL as seropositivity, suggesting a recent pertussis infection within the past year in the absence of pertussis vaccination, and PT-IgG concentration of ≥ 100 IU/mL was considered diagnostic evidence of an acute or recent infection if the individual had not received a booster dose of the pertussis vaccine within the preceding year. PT-IgG concentration < 40 IU/mL indicated no evidence for a previous infection of *Bordetella pertussis*.

### Reported incidence of pertussis

Data on pertussis cases were obtained from the National Notifiable Disease Reporting System (NNDRS). To calculate the incidence rate of pertussis, we divided the number of reported cases by the total population, with the rate expressed per 100,000 individuals. The population figures used as the denominator in these calculations were sourced from the Shandong Statistical Yearbook [12]. From 2017 to 2023, the population of Shandong Province maintained a relatively stable level, fluctuating between 101 million and 102 million.

### Estimated incidence of pertussis

The incidence rate of pertussis infection was estimated using the method described in established literature [11]. It was observed that following infection, the PT-IgG concentration typically takes 58.6 days to drop to a level of 100 IU/mL and 365 days (12 months) to reach a level of 30 IU/mL. With the threshold of 100 IU/mL, the incidence of infection was calculated using the formula: 365.2/58.6 × the proportion of subjects with PT-IgG concentration at or above 100 IU/mL. To ensure the accuracy of incidence assessment, it was essential to consider the potential interference of recent vaccination with pertussis-containing vaccines. Therefore, the incidence estimation excluded individuals who had received such vaccinations within the past 12 months. Taking into account the current immunization schedule in China, which includes 4 doses of pertussis vaccine administered at 3, 4, 5, and 18–24 months of age, the incidence estimation of pertussis infection in this study was confined to individuals aged three years and above.

### Statistical analysis

Data analysis was conducted utilizing Microsoft Excel 2020 and IBM SPSS Statistics 26 software. Quantitative data were summarized using means and their associated 95% Confidence Intervals (CI), and qualitative data were presented as percentages. The chi-squared test was used to assess variations in rates or composition ratios among different time periods, regions, age groups, and genders within the population. A significant level of *p* < 0.05 was used to determined statistical significance. The Spearman correlation coefficient r was utilized to evaluate the linear correlation relationship between the positive rate of PT-IgG antibodies and the reported pertussis incidence across various age groups and regions.

## Results

Since the introduction of DTP vaccine into EPI in China, there has been a significant decrease in the incidence of pertussis nationwide. In Shandong Province, the reported pertussis incidence plummeted from a peak of 361.95 per 100,000 individuals in 1973 (245,808 cases) to a low level of 0.10 per 100,000 individuals in 2006 (94 cases). In recent years, there has been a noticeable upward trend in the number of pertussis cases. In 2018, the incidence reached its highest point in nearly 30 years, with a reported incidence of 5.77 per 100,000 individuals (5,770 cases). In this study, a total of 9,238 blood specimens were collected between 2017 and 2023. Analysis of the specimens indicated that 8.05% (95% CI: 7.50%~8.60%) of the cases tested positive for PT-IgG, and 2.66% (95% CI: 2.33%~2.99%) identified as acute or recent pertussis infections. The majority of the specimens showed negative results, with 48.81% (95% CI: 47.79%~49.83%) of the total specimens falling below the lower detection limit (Table 1).

**Table 1:** Pertussis serum antibodies surveillance in community-based populations in Shandong Province, 2017 ~ 2023

Variables	*N*	*N*. of surveyed subjects (%, 95%CI) in concentrations of PT-IgG				χ2	*p*-value
		< 5 IU/mL	5 ~ < 40 IU/mL	40 ~ < 100 IU/mL	≥ 100 IU/mL		
Year							
2017	1138	575 (50.53, 47.63 ~ 53.43)	484 (42.53, 39.66 ~ 45.40)	55 (4.83, 3.58 ~ 6.08)	24 (2.11, 1.27 ~ 2.95)	23.909^a^ 31.566^b^	< 0.001^a^ < 0.001^b^
2018	1287	496 (38.54, 35.88 ~ 41.20)	681 (52.91, 50.18 ~ 55.64)	74 (5.75, 4.48 ~ 7.02)	36 (2.80, 1.90 ~ 3.70)		
2019	1607	797 (49.60, 47.16 ~ 52.04)	638 (39.70, 37.31 ~ 42.09)	105 (6.53, 5.32 ~ 7.74)	67 (4.17, 3.19 ~ 5.15)		
2020	1266	742 (58.61, 55.90 ~ 61.32)	444 (35.07, 32.44 ~ 37.70)	64 (5.06, 3.85 ~ 6.27)	16(1.26, 0.65 ~ 1.87)		
2021	1486	729 (49.06, 46.52 ~ 51.60)	644 (43.34, 40.82 ~ 45.86)	85 (5.72, 4.54 ~ 6.90)	28 (1.88, 1.19 ~ 2.57)		
2022	1244	514 (41.32, 38.58 ~ 44.06)	629 (50.56, 47.78 ~ 53.34)	58 (4.66, 3.49 ~ 5.83)	43 (3.46, 2.44 ~ 4.48)		
2023	1210	656 (54.21, 51.40 ~ 57.02)	465 (38.43, 35.69 ~ 41.17)	57 (4.71, 3.52 ~ 5.90)	32 (2.64, 1.74 ~ 3.54)		
Age group (years)							
<3	1536	667 (43.42, 40.94 ~ 45.90)	693 (45.12, 42.63 ~ 47.61)	113 (7.36, 6.05 ~ 8.67)	63 (4.10, 3.11 ~ 5.09)	43.098^a^ 26.214^b^	< 0.001^a^ < 0.001^b^
3 ~ 5	1573	878 (55.82, 53.37 ~ 58.27)	610 (38.78, 36.37 ~ 41.19)	58 (3.69, 2.76 ~ 4.62)	27 (1.72, 1.08 ~ 2.36)		
6 ~ 12	1643	883 (53.74, 51.33 ~ 56.15)	645 (39.26, 36.90 ~ 41.62)	87 (5.30, 4.22 ~ 6.38)	28 (1.70, 1.07 ~ 2.33)		
13 ~ 16	1511	719 (47.58, 45.06 ~ 50.10)	664 (43.94, 41.44 ~ 46.44)	82 (5.43, 4.29 ~ 6.57)	46 (3.04, 2.17 ~ 3.91)		
17 ~ 19	1407	668 (47.48, 44.87 ~ 50.09)	617(43.85, 41.26 ~ 46.44)	89 (6.33, 5.06 ~ 7.60)	33 (2.35, 1.56 ~ 3.14)		
≥ 20	1568	694 (44.26, 41.80 ~ 46.72)	756 (48.21, 45.74 ~ 50.68)	69 (4.40, 3.38 ~ 5.42)	49 (3.13, 2.27 ~ 3.99)		
Gender							
Male	4742	2284 (48.17, 46.75 ~ 49.59)	2068 (43.61, 42.20 ~ 45.02)	262 (5.53, 4.88 ~ 6.18)	128 (2.70, 2.24 ~ 3.16)	0.383^a^ 0.050^b^	0.536^a^ 0.824^b^
Female	4496	2225 (49.49, 48.03 ~ 50.95)	1917 (42.64, 41.19 ~ 44.09)	236 (5.25, 4.60 ~ 5.90)	118 (2.62, 2.15 ~ 3.09)		
Region							
Eastern	2734	1475 (53.95, 52.08 ~ 55.82)	1062 (38.84, 37.01 ~ 40.67)	134 (4.90, 4.09 ~ 5.71)	63(2.30, 1.74 ~ 2.86)	11.804^a^ 2.996^b^	0.003^a^ 0.224^b^
Central	2961	1293 (43.67, 41.88 ~ 45.46)	1388(46.88, 45.08 ~ 48.68)	190 (6.42, 5.54 ~ 7.30)	90 (3.04, 2.42 ~ 3.66)		
Western	3543	1741 (49.14, 47.34 ~ 50.94)	1535 (43.32, 41.54 ~ 45.10)	174 (4.91, 4.13 ~ 5.69)	93 (2.62, 2.04 ~ 3.20)		
Total	9238	4509 (48.81, 47.79 ~ 49.83)	3985 (43.14, 42.13 ~ 44.15)	498 (5.39, 4.93 ~ 5.85)	246 (2.66, 2.33 ~ 2.99)		

^a^Comparison of antibody positivity rates

^b^Comparison of acute or recent infection rates

### Time correlation

Analysis of the positivity rates for PT-IgG antibodies and the rates of recent infections revealed that both reached their peak in 2019, with 10.70% (95% CI: 9.19%~12.21%) and 4.17% (95% CI: 3.19%~5.15%), respectively. A significant decline was observed in 2020 and 2021 thereafter. There were significant differences in both rates across the years (χ^2^ = 23.909, *p* < 0.001; χ^2^ = 31.566, *p* < 0.001) (Table 1).

**Fig. 1:**
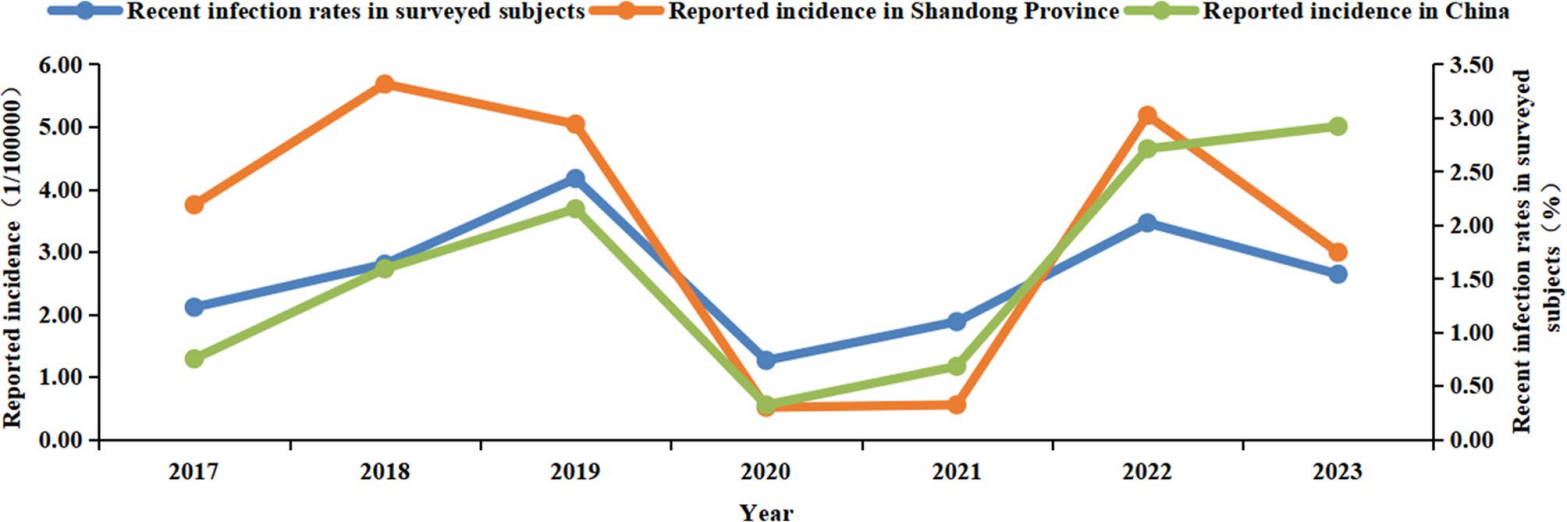
Distribution of recent infection rate and reported incidence of pertussis by year, 2017 ~ 2023

From 2017 to 2023, a total of 155,979 pertussis cases were reported in China, with the reported incidence rate (per 100,000 individuals, the same below) of 0.75 (10,390 cases), 1.59 (22,057 cases), 2.15 (30,027 cases), 0.32 (4,475 cases), 0.68 (9,611 cases), 2.71 (38,295 cases), and 2.92 (41124 cases) for each corresponding year. During the same period in Shandong Province, 23,940 cases were reported, accounting for 15.35% of the national total. The reported incidence rates varied from 0.51 to 5.68, with the rates of 3.75 (3,729 cases), 5.68 (5,770 cases), 5.04 (5,063 cases), 0.51 (516 cases), 0.55 (563 cases), 5.18 (5,263 cases), and 2.99 (3,036 cases) for each year respectively. The rate of recent pertussis infections among the community-based population in Shandong Province displayed a fluctuating upward trend, which showed strong linear correlations with the reported incidence rate of pertussis in Shandong Province (*r* = 0.821, *p* = 0.023) and across the nation (*r* = 0.750, *p* = 0.052) for the corresponding year (Table 1; Fig. 1).

## Population distribution

The age of the surveyed subjects ranged from 2 months to 81 years, with a male-to-female ratio of 1.05:1 (4,742 to 4,496). The median ages of the six groups (< 3, 3 ~ 5, 6 ~ 12, 13 ~ 16, 17 ~ 19, and ≥ 20 years) were 2.0, 4.4, 9.1, 14.6, 18.1, and 37.6 years, respectively. The lowest seropositivity rate was noted in the 3 ~ 5 years old group (5.40%, 95% CI: 4.28%~6.52%), while the highest rate was observed in the under 3 years old group (11.46%, 95% CI: 9.87%~13.05%). The recent infection rate plummeted from a peak of 4.10% (95% CI: 3.11%~5.09%) in the under 3 years old group to the nadir in the 6 ~ 12 years old group (1.70%, 95% CI: 1.07%~2.33%), after which it gradually rose with increasing age. Significant differences in antibody positivity rates (χ^2^ = 43.098, *p* < 0.001) and recent infection rates (χ^2^ = 26.214, *p* < 0.001) were observed across the various age groups (Table 1).

As depicted in Table 2, there was a markedly decreasing trend with age in the incidence of pertussis reported in Shandong Province from 2017 to 2023. This reported incidence demonstrated an extremely weak correlation with the recent infection rates observed among community-based subjects (*r* = 0.086, *p* = 0.872).

**Table 2:** Distribution of recent infection rates among community-based populations and reported incidence of pertussis at different age groups in Shandong Province, 2017 ~ 2023

Year	< 3 years		3 ~ 5 years		6 ~ 12 years		13 ~ 16 years		17 ~ 19 years		≥ 20 years			
	*N*. of surveyed subjects (recent infection rates%, 95%CI)	Reported incidence(*n*/100000)	*N*. of surveyed subjects (recent infection rates%, 95%CI)	Reported incidence(*n*/100000)	*N*. of surveyed subjects (recent infection rates%, 95%CI)	Reported incidence(*n*/100000)	*N*. of surveyed subjects (recent infection rates%, 95%CI)	Reported incidence(*n*/100000)	*N*. of surveyed subjects (recent infection rates%, 95%CI)	Reported incidence(*n*/100000)	*N*. of surveyed subjects (recent infection rates%, 95%CI)	Reported incidence(*n*/100000)	*r*	*p*-value
2017	192 (2.60, 0.35 ~ 4.85)	84.03	192 (2.08, 0.06 ~ 4.10)	12.92	200 (0.50, 0.00 ~ 1.48)	3.45	178 (1.69, 0.00 ~ 3.58)	0.17	166 (3.61, 0.77 ~ 6.45)	0.00	210 (2.38, 0.32 ~ 4.44)	0.11	−0.257	0.623
2018	210 (3.33, 0.90 ~ 5.76)	125.67	219 (1.83, 0.05 ~ 3.61)	23.97	226 (0.88, 0.00 ~ 2.10)	5.47	207 (3.86, 1.24 ~ 6.48)	0.11	207 (3.86, 1.24 ~ 6.48)	0.07	218 (3.21, 0.87 ~ 5.55)	0.12	−0.580	0.228
2019	256 (5.47, 2.68 ~ 8.26)	102.59	295 (2.71, 0.86 ~ 4.56)	23.99	315 (2.86, 1.02 ~ 4.70)	5.87	254 (7.87, 4.56 ~ 11.18)	0.35	234 (3.42, 1.09 ~ 5.75)	0.00	253 (3.16, 1.00 ~ 5.32)	0.12	−0.143	0.787
2020	199 (3.520.96 ~ 6.08)	7.87	217 (0.46, 0.00 ~ 1.36)	3.59	210 (0.48, 0.00 ~ 1.41)	0.76	216 (0.00, 0.00 ~ 0.00)	0.07	185 (1.08, 0.00 ~ 2.57)	0.07	239 (2.09, 0.28 ~ 3.90)	0.03	0.116	0.827
2021	282 (2.13, 0.44 ~ 3.82)	6.46	246 (0.81, 0.00 ~ 1.93)	3.74	250 (2.40, 0.50 ~ 4.30)	1.83	238 (0.42, 0.00 ~ 1.24)	0.13	224 (1.34, 0.00 ~ 2.85)	0.00	246 (4.07, 1.60 ~ 6.54)	0.02	−0.086	0.872
2022	198 (6.57, 3.12 ~ 10.02)	48.43	202 (1.98, 0.06 ~ 3.90)	37.83	231 (1.30, 0.00 ~ 2.76)	20.39	214 (3.74, 1.20 ~ 6.28)	0.94	201 (1.49, 0.00 ~ 3.16)	0.11	198 (6.06, 2.74 ~ 9.38)	0.14	0.314	0.544
2023	199 (5.53, 2.35 ~ 8.71)	23.21	202 (1.98, 0.06 ~ 3.90)	12.30	211 (2.84, 0.60 ~ 5.08)	17.98	204 (2.94, 0.62 ~ 5.26)	1.18	190 (1.58, 0.00 ~ 3.35)	0.11	204 (0.98, 0.00 ~ 2.33)	0.07	0.829	0.042
Total	1536 (4.10, 3.11 ~ 5.09)	56.89	1573 (1.72, 1.08 ~ 2.36)	16.90	1643 (1.70, 1.07 ~ 2.33)	7.96	1511 (3.04, 2.17 ~ 3.91)	0.42	1407 (2.35, 1.56 ~ 3.14)	0.05	1568 (3.13, 2.27 ~ 3.99)	0.09	0.086	0.872

### Regional distribution

The eastern region exhibited the lowest antibody positivity rate at 7.21% (95% CI: 6.24%~8.18%), while the central region reported a relatively higher rate of 9.46% (95% CI: 8.41%~10.51%). Recent infection rates across the eastern (2.30%, 95% CI: 1.74%~2.86%), central (3.04%, 95% CI: 2.42%~3.66%) and western (2.62%, 95% CI: 2.04%~3.20%) regions did not show significant differences (χ^2^ = 2.996, *p* = 0.224). Within each region, there were moderate or strong correlations observed between the recent infection rate of pertussis among the community-based populations and the reported incidence rate (Table 3).

**Table 3:** Distribution of recent infection rates among community-based populations and reported incidence of pertussis at different regions in Shandong Province, 2017 ~ 2023

Year	Eastern				Central				Western			
	N. of surveyed subjects (recent infection rates%, 95%CI)	*Reported incidence *(*n*/100000)	*r*	*p*-value	N. of surveyed subjects (recent infection rates%, 95%CI)	Reported incidence (*n*/100000)	*r*	*p*-value	N. of surveyed subjects (recent infection rates%, 95%CI)	*Reported incidence *(*n*/100000)	*r*	*p-value*
2017	600 (1.67, 0.64 ~ 2.70)	0.54	0.487	0.268	300 (3.33, 1.30 ~ 5.36)	7.00	0.500	0.253	238 (1.68, 0.05 ~ 3.31)	3.49	0.786	0.036
2018	300 (3.33, 1.30 ~ 5.36)	1.20			463 (2.38, 0.99 ~ 3.77)	9.28			524 (2.86, 1.43 ~ 4.29)	6.46		
2019	313 (1.60, 0.21 ~ 2.99)	0.99			660 (5.00, 3.34 ~ 6.66)	8.51			634 (4.57, 2.94 ~ 6.20)	5.39		
2020	300 (2.00, 0.42 ~ 3.58)	0.16			360 (1.39, 0.18 ~ 2.60)	0.96			606(0.83, 0.11 ~ 1.55)	0.42		
2021	300 (2.00, 0.42 ~ 3.58)	0.17			578 (1.90, 0.79 ~ 3.01)	0.61			608 (1.81, 0.75 ~ 2.87)	0.85		
2022	321 (3.12, 1.22 ~ 5.02)	1.68			300 (3.67, 1.54 ~ 5.80)	5.35			623 (3.53, 2.08 ~ 4.98)	8.15		
2023	600 (2.67, 1.38 ~ 3.96)	0.62			300 (3.00, 1.07 ~ 4.93)	6.63			310 (2.26, 0.61 ~ 3.91)	1.85		
Total	2734 (2.30, 1.74 ~ 2.86)	0.76			2961 (3.04, 2.42 ~ 3.66)	5.48			3543 (2.62, 2.04 ~ 3.20)	3.80		

### Estimated infection rate of pertussis

Table 4 presents the estimated infection rates for 7,702 individuals aged over 3 years between 2017 and 2023, with an average rate of 14,807 per 100,000 individuals. The estimated infection rate was the lowest for the 3 ~ 12 years age group and then increased sharply in the age group of 13 ~ 16 years (18,973 per 100,000 individuals). In the ≥ 20 years group, a further increase in the infection rate up to 19,475 per 100,000 individuals was observed. The estimated infection rates were considerably higher for all age groups compared to the incidence of reported cases, with the incidence exceeding the reported rates by up to 29,2340.00-fold in the 17 ~ 19 years group and 216,388.89-fold in the group aged 20 years and above. Significant differences were also noted in the distribution across different years and regions, with the largest exceedance observed in 2021 (31633.33-fold) and in the eastern region (31,544.44-fold), respectively.

**Table 4:** Estimated infection rate and reported incidence of pertussis among populations aged ≥ 3 years in Shandong Province, 2017 ~ 2023

Variables	*N*. of surveyed subjects	*N*. of PT-IgG ≥ 100 IU/mL	Estimated infection rate (95%CI) (*n*/100000)	Reported incidence (*n*/100000)	Excess Incidence*
Years					
2017	946	19	12 517(10 408 ~ 14 626)	1.00	12517.00
2018	1077	29	16 781(14 549 ~ 19 013)	1.69	9929.59
2019	1351	53	24 449(22 157 ~ 26 740)	1.74	14051.15
2020	1067	9	5 257(3 918 ~ 6 596)	0.26	20219.23
2021	1204	22	11 388(9 593 ~ 13 182)	0.36	31633.33
2022	1046	30	17 874(15 552 ~ 20 196)	3.75	4766.40
2023	1011	21	12 945(10 876 ~ 15 014)	2.32	5579.74
Age group (years)					
3 ~ 5	1573	27	10 697(9 170 ~ 12 225)	16.90	632.96
6 ~ 12	1643	28	10 621(9 131 ~ 12 111)	7.96	1334.30
13 ~ 16	1511	46	18 973(16 996 ~ 20 950)	0.42	45173.81
17 ~ 19	1407	33	14 617(12 771 ~ 16 463)	0.05	292340.00
≥ 20	1568	49	19 475(17 515 ~ 21 435)	0.09	216388.89
Region					
Eastern	2305	42	11 356(10 060 ~ 12 651)	0.36	31544.44
Central	2437	72	18 412(16 874 ~ 19 951)	2.61	7054.41
Western	2960	69	14 527(13 258 ~ 15 797)	1.78	8161.24
Total	7702	183	14 807(14 014 ~ 15 601)	1.59	9312.58

*Exceeded times of estimated infection rate over the reported incidence

## Discussion

The number of reported pertussis cases in Shandong Province experienced a notable increase from 2008 to 2023, especially after the province wide surveillance of pertussis was strengthened in 2016. Compared to 2017 ~ 2019, broad declines were observed both in the reported incidence and estimated infection rate of pertussis shortly after the COVID-19 pandemic surge in 2020. Since 2022, abrupt surges occurred and continued up to now. The reported incidence rate in 2022 (5.18/100,000) was 46 times higher than that in 2008 (0.11/100,000), marking a pronounced trend known as the “reappearance of pertussis” [7].

PT-IgG is a pertussis-specific antibody, and a single serum sample with a concentration exceeding the defined threshold is generally accepted as an indicator of recent infection or vaccination within the past 12 months [13]. This serological marker has been utilized in many countries to characterize the epidemiology of pertussis and to estimate the infection rate within populations [11, 14–16]. Considering the current immunization program for pertussis in China, the antibody concentrations in young children are likely influenced by both vaccination and natural infection. Given that PT-IgG concentration tend to decrease markedly following a pertussis infection for one year, surveillance data among individuals aged ≥ 3 years can be used to estimate the clinical infection rate rather than the impact of vaccination. Our surveillance data revealed a very strong linear correlation between the seropositive rate of pertussis in community-based populations and the reported incidence rate in terms of temporal distribution. However, the infection rate estimated from serological results markedly exceeds the reported rate. This significant discrepancy suggests that the actual infection rates of pertussis in Shandong Province substantially exceed those reported by physicians based on clinical findings. Similar observations have been reported in other regions within China. Studies conducted in Beijing, Chongqing and Tianjin have determined the infection rates exceeding 6,000 per 100,000 individuals [17], 7,290 per 100,000 individuals [18], and 10,852 per 100,000 individuals [6], respectively.

Several factors account for the substantial discrepancies between reported pertussis incidence and the estimated infection rate. Firstly, the current case reporting system in China predominantly depends on clinical symptoms, which may result in underreporting of pertussis, as some general practitioners lack the requisite attention and awareness to identify the disease, particularly in instances of mild cases, atypical presentations, and complex infections with multiple pathogens. Secondly, advancements in pertussis testing, such as the introduction of PCR and ELISA, have facilitated the identification of cases and raised the awareness among general practitioners [19, 20]. However, these diagnostic methods have not been widely adopted in medical institutions in China, primarily due to the absence of national diagnostic standards and lack of uniform criteria for interpreting test results across different countries. A survey assessing the laboratory diagnostic capacity for pertussis in Grade Ⅱ and Ⅲ medical institutions across Shandong Province in 2018 revealed that, just 3.7% of these institutions had established laboratory testing for pertussis, and the eastern region of the province was notably deficient in testing capacity when compared to the central and western regions [21]. Our study indicates that there is no significant variation in the recent infection rate among community-based populations across the different regions, while it is noteworthy that the reported incidence in the eastern part is markedly lower compared to the other two regions. This disparity suggests that the lack of laboratory diagnosis may be a significant factor contributing to the under recognition of pertussis. Furthermore, adolescents and adults infected with pertussis often exhibit asymptomatic, mild or persistent coughing [22], which may result in the neglect of seeking medical treatment. This can severely impede the identification and control of cases, ultimately exacerbate the spread of the disease. In China, the reported cases of pertussis were predominantly found in infants and young children, with a substantial proportion occurring in those under 3 years of age. From 2011 to 2017, this age group constituted 82.46% of the reported pertussis cases [23], and decreased to 72.31% from 2018 to 2021 [24]. In recent years, some countries have reported a rapid increase in pertussis cases among adolescents and adults, such as in the European Union, individuals over 15 years old accounted for 62% of the total number of reported cases in 2018 [25]. This shift highlights the underreporting of pertussis among middle-aged individuals, which is a concerning issue as it suggests that the true burden of the disease in this demographic may be significantly higher than reported [26]. Our evidence corroborates this claim, showing that the estimated infection rate among community-based populations begins to rise from the age of 13 and peaks in adults aged 20 and older.

There are some limitations in this study. First, the findings may not be fully applicable to the entire population in Shandong Province, as only three variables were available for the samples (age, sex and region), and no extensive statistical analysis could be performed to compare these sociodemographic variables with the broader demographics of the country. Secondly, the limited sample size may have reduced the accuracy of statistical analysis, and further investigation is needed particularly for individuals over 20 years old, to elucidate the trend of antibody levels in relation to age. Furthermore, the threshold for antibody levels indicating pertussis infection, as used in our study for estimating incidence, was derived from consultations with previous European studies. In consideration of distinct situation of pertussis prevalence and immunization pattern, further efforts should be made to develop a local definition for serum antibody of pertussis and validate the existing ELISA methods available in China.

## Conclusion

Our analysis has brought to light several critical issues. First, the estimated incidence of pertussis infections across all age groups substantially surpasses the rates reported by physicians based on clinical diagnosis. This underscores the inadequacy of the current surveillance system, and emphasizes the value of seroepidemiology as a complementary tool to enhance disease surveillance in conjunction with existing programs. Secondly, there is a notable discrepancy in the age-specific profile between the reported incidence and the estimated infection among community-based population. The highest reported prevalence, characterized as hyper-endemic, is observed among young children under the age of 6, while the infection rates detected among the population increased with age and reached a peak in individuals over 20 years old. This suggests a need to focus on surveillance efforts and consider the implementation of additional booster immunizations for adolescents and adults. Furthermore, the utilization of laboratory confirmation through serological testing has emerged as pivotal diagnostic methods, which offer great sensitivity and rapid results for case diagnosis and assessing the infection rates within the population.

## Supplementary Information

Supplementary Material 1.

## Data Availability

Data is provided within the manuscript or supplementary information files.

## Abbreviations

PT-IgG: pertussis toxin IgG
EPI: Expanded Programme on Immunization
NNDRS: National Notifiable Disease Reporting System
