# Supplementary material for: Correlation analysis between positivity rate of immunoglobulin G antibodies against pertussis toxin among community-based populations and reported pertussis incidence in Shandong, China: a seven-year seroepidemiological study
Source: BMC Infect Dis. 2025 Oct 24;25:1404. doi: 10.1186/s12879-025-11802-9 (PMC12551178; doi:10.1186/s12879-025-11802-9)
Supplement: Supplementary file 1 — Supplementary Material 1. [file 12879_2025_11802_MOESM1_ESM.zip › Questionnaire.docx]

**Questionnaire of seroepidemiological Survey**

Survey location： （City） （county） （town/street）

Investigator: Investigation time:

**Basic information**

1. Name: (Name of guardian for individuals under 18 years old: )
2. Gender: □ Male □ Female
3. Date of Birth:
4. Contact phone number:
5. Occupation: □Scattered children □Preschool children □Student □Teacher

□Medical staff □Official staff □Worker □Farmer □Retired personnel

□Household or unemployment □Others:

6. Do you have symptoms of respiratory infection in the past three months? □ Yes □ No

7. Do you have used antibiotics in the past three months? □ Yes □ No

8. Have you lived in the survey location for at least 12 months? □ Yes □ No

**Vaccination history**

9. Have you received the vaccine containing pertussis antigen components? □ Yes □ No

10. History of pertussis vaccination

A total of _________ doses of pertussis vaccination:

| Dose | Vaccination date | Vaccine type and manufacturer |
| --- | --- | --- |
| The first dose |  |  |
| The second dose |  |  |
| The third dose |  |  |
| The fourth dose |  |  |
| Supplement |  |  |

**Thank you for your cooperation.**
