# Supplementary material for: Correlation analysis between positivity rate of immunoglobulin G antibodies against pertussis toxin among community-based populations and reported pertussis incidence in Shandong, China: a seven-year seroepidemiological study
Source: BMC Infect Dis. 2025 Oct 24;25:1404. doi: 10.1186/s12879-025-11802-9 (PMC12551178; doi:10.1186/s12879-025-11802-9)
Supplement: Supplementary file 1 — Supplementary Material 1. [file 12879_2025_11802_MOESM1_ESM.zip › Questionnaire.pdf]

Questionnaire of seroepidemiological Survey

Survey location: _________ (City) _________ (county) ___________ (town/street)

Investigator:____________ Investigation time:_____________

Basic information

1. Name: ________(Name of guardian for individuals under 18 years old: ________)
2. Gender: ☐ Male ☐ Female
3. Date of Birth: ____________
4. Contact phone number: __________
5. Occupation: ☐Scattered children ☐Preschool children ☐Student ☐Teacher

   ☐Medical staff ☐Official staff ☐Worker ☐Farmer ☐Retired personnel

   ☐Household or unemployment ☐Others: __________

6. Do you have symptoms of respiratory infection in the past three months? ☐ Yes ☐ No

7. Do you have used antibiotics in the past three months? ☐ Yes ☐ No

8. Have you lived in the survey location for at least 12 months? ☐ Yes ☐ No

Vaccination history

9. Have you received the vaccine containing pertussis antigen components? ☐ Yes ☐ No

10. History of pertussis vaccination

A total of _________ doses of pertussis vaccination:

Dose	Vaccination date	Vaccine type and manufacturer
The first dose		
The second dose		
The third dose		
The fourth dose		
Supplement		

Thank you for your cooperation.
